# Willingness and ability to pay for artemisinin-based combination therapy in rural Tanzania

**DOI:** 10.1186/1475-2875-7-227

**Published:** 2008-10-31

**Authors:** Eleonor C Saulo, Birger C Forsberg, Zul Premji, Scott M Montgomery, Anders Björkman

**Affiliations:** 1 Malaria Research Unit (M9), Division of Infectious Diseases, Department of Medicine, Karolinska University Hospital, 171 76 Stockholm, Sweden; 2 Division of International Health (IHCAR), Department of Public Health Sciences, Karolinska Institutet, 171 77 Stockholm, Sweden; 3 Department of Parasitology and Entomology, Muhimbili University College of Health Sciences, PO Box 65001, Dar es Salaam, United Republic of Tanzania; 4 Clinical Epidemiology Unit, Department of Medicine, Karolinska University Hospital, Karolinska Institutet, 171 76 Stockholm, Sweden

## Abstract

**Background:**

The aim of this study was to analyse willingness to pay (WTP) and ability to pay (ATP) for ACT for children below five years of age in a rural setting in Tanzania before the introduction of artemisinin-based combination therapy (ACT) as first-line treatment for uncomplicated malaria. Socio-economic factors associated with WTP and expectations on anti-malaria drugs, including ACT, were also explored.

**Methods:**

Structured interviews and focus group discussions were held with mothers, household heads, health-care workers and village leaders in Ishozi, Gera and Ishunju wards in north-west Tanzania in 2004. Contingent valuation method (CVM) was used with "take-it-or-leave-it" as the eliciting method, expressed as WTP for a full course of ACT for a child and households' opportunity cost of ACT was used to assess ATP. The study included descriptive analyses with multivariate adjustment for potential confounding factors.

**Results:**

Among 265 mothers and household heads, 244 (92%, CI = 88%–95%) were willing to pay Tanzanian Shillings (TSh) 500 (US$ 0.46) for a child's dose of ACT, but only 55% (49%–61%) were willing to pay more than TSh 500. Mothers were more often willing to pay than male household heads (adjusted odds ratio = 2.1, CI = 1.2–3.6). Socio-economic status had no significant effect on WTP. The median annual non-subsidized ACT cost for clinical malaria episodes in an average household was calculated as US$ 6.0, which would represent 0.9% of the average total consumption expenditures as estimated from official data in 2001. The cost of non-subsidized ACT represented 7.0% of reported total annual expenditure on food and 33.0% of total annual expenditure on health care.

"Rapid effect," "no adverse effect" and "inexpensive" were the most desired features of an anti-malarial drug.

**Conclusion:**

WTP for ACT in this study was less than its real cost and a subsidy is, therefore, needed to enable its equitable affordability. The decision taken in Tanzania to subsidize Coartem^® ^fully at governmental health care facilities and at a consumer price of TSh 300–500 (US$ 0.28–0.46) at special designated shops through the programme of Accredited Drug Dispensing Outlets (ADDOs) appears to be well founded.

## Background

Malaria constitutes a major burden on individuals and on the community in low-income countries. In Tanzania, it has been estimated that 1% of GDP is spent on the disease. For comparison, total health expenditure in Tanzania constitutes about 4% of GDP. 71% of the total expenditure on malaria is from private sources [1].

In sub-Saharan Africa, the utilization of ineffective drugs in recent years has led to treatment failures and elevated rates of mortality, particularly among young children [2,3]. Artemisinin-based combination therapy (ACT) is, therefore, recommended as the first-line anti-malarial treatment strategy in most parts of sub-Saharan Africa [4]. Efficacy and effectiveness studies have shown that ACT provides cure rates of over 90% [5-7] and generally, it is also well tolerated [8,9]. However, good quality of health care is critical to ensure efficacy and prevent parasitic resistance to the anti-malarial drugs, by limiting over-treatment and unnecessary drug exposure, as well as ensuring compliance to standard treatment doses.

The cost of artemisinin derivatives is a limiting factor for the affordability and optimal use of ACT. Various forms of ACT are 5–10-fold more costly compared with the previously recommended treatments, chloroquine or sulphadoxine/pyrimethamine (SP). Coartem^® ^(artemether-lumefantrine), a co-formulated ACT, was announced in 2006 to be available at a reduced public market price at an average of US$ 1.0 per adult/child treatment course [10]. Another co-formulated ACT consisting of artesunate and amodiaquine (ART-AQ) was announced in 2007 to be available at a market price below US$ 0.5 per child treatment and US$ 1.0 per adult treatment [11]. Access and affordability are critical for optimal compliance to the new treatment strategy and an important issue is how much people are willing and able to pay for ACT. Studies on willingness to pay (WTP) and ability to pay (ATP) for ACT are needed to guide financial strategies to ensure access to recommended malaria treatments for all.

Two studies on WTP for malaria combination therapies have been published from Africa [12,13]. They assessed WTP among household heads [12] and mothers [13] respectively, but did not explore ATP. Both studies concluded that the WTP and the actual market price of ACT diverge and that subsidies are needed.

The present study estimates and compares WTP for combination therapy among both mothers and household heads and provides information on ATP from household heads in an area not previously reached by ACT at the time of the study (2004). The study also compares annual household expenditures on ACT by using different cost scenarios and relates them to WTP and to official data on total annual rural household consumption expenditure in Tanzania as well as to reported household expenditures in the study area. Perceptions of anti-malarial drugs and the treatment-decision process in the family are also explored.

## Methods

### Study site

The study was conducted in Ishozi, Gera and Ishunju wards (small administrative units) in Missenye District in Kagera Region in April 2004. The district was selected by convenience. It is a rural district and it is well representative of the region, which is mainly agricultural. Coffee is a main cash crop and the staple food is plantain and beans. Parts of the region are difficult to access due to poor roads and many rural parts lack electricity. The wards are situated at an altitude of about 1200 meters above sea level and have a total population of 15 000. There are 11 villages in the three wards. The nearest town, Bukoba, is 20–30 km away. At the time of the data collection in 2004, the fare of traveling to Bukoba by public transport was Tanzanian Shillings (TSh) 500 to 1000 (US$ 0.46–0.92), depending on the villages' location.

The vast majority of the people in the area are peasants. At the time of the study the price per kilo of coffee was TSh 200 (US$ 0.18). During the past decade the market price of coffee had decreased substantially and, therefore, the availability of cash had declined. The agricultural yield in general had also gone down. Overall, income had decreased and expenses increased in the region. Except for coffee, crops are mainly used for home consumption.

There are eight dispensaries in the area: two government-run, one private and five run by Patage, a Non-Governmental Organization (NGO). Children get free services at the government health care facilities. The NGO supports orphans with free health care and education. All other services are charged for. There is one private pharmacy in the area, placed in Ishozi ward. The pharmacy receives about 50 customers per week. There are also a number of "unofficial" drug vendors in the region.

Malaria transmission occurs throughout the year but with peak transmission during and after the rainy seasons, which commonly occur March to May and October to December [14].

ACT was largely unknown to the local people at the time of the study. SP was the first-line malaria treatment and was well known to the people. Fansidar, the most common brand of SP, could be bought at a price from TSh 200 (US$ 0.18) per child treatment dose. The only artemisinin derivate available was Artemedine^® ^(artemether), produced in China. This monotherapy could be bought in Bukoba at TSh 6000 (US$ 5.51) per package containing 12 tablets of 50 mg.

### Qualitative data collection

Two focus group discussions (FGD) were conducted in Nyarugongo village in Ishozi ward. One FGD included seven men (25–45 years old) whereas the other included eight women (25–45 years old). The FGDs provided opinions and perceptions on malaria, its treatment and the need for new anti-malarial drugs, which were used when designing the questionnaires for the quantitative data collection.

In-depth interviews were held with village leaders from four out of five villages that were later selected for the quantitative data collection. One village leader was not available. The four village leaders were all men and had been in their position from 4 to 24 years.

In-depth interviews were also conducted with four health-care workers at four different dispensaries, two governmental and two run by the NGO. Two were nurses, one a midwife and one a clinical assistant. The pharmacist in the pharmacy was also interviewed. The drug vendors, who have continued to sell chloroquine after its official withdrawal from the market in 2001, were not willing to participate in this study, since they were reluctant to answer questions about their activities.

### Quantitative data collection

The data collection took place in April 2004 during the peak season for malaria transmission. By purposive selection on the basis of their accessibility and representativeness of the target population, the villages Kashaka, Kashambya, Kashekya, Katano and Ishunju were selected to represent the three wards. In each village 25–35 mothers of children under five years of age and 25–35 heads of households with children under five years of age were targeted depending on the size of the village. By starting in the village center and proceeding randomly from household to household towards the periphery of the village and by alternating the type of respondent for each household, a total number of 135 mothers and 134 heads of different households were included in the study. Only one respondent, either a mother of a child under five years of age or the financial head, was randomly selected from each household.

Two different questionnaires in the national language Kiswahili were used, one for mothers and one for household heads. The local language Kihaya was used when it was needed to clarify the questions to the respondents. Most questions were close-ended. Questions covered household characteristics, willingness to pay for malaria treatment for children as explained below, knowledge on and perceptions of malaria, decision-making processes in the household and preferences of characteristics of new anti-malarial drugs. The term "homa ya malaria" was used when referring to malaria and the term was defined according to the local perception of the disease. Household characteristics included household size and composition, education and occupation of the respondents and household expenditures.

Contingent valuation method (CVM), a survey-based method with hypothetical situations to determine individuals' monetary valuation of health care or health states, was used to elicit WTP [15-17]. "Take it or leave it" (TIOLI) was used as eliciting method.

To assess WTP for ACT, a scenario was demonstrated wherein a child in the household is suffering from malaria and the interviewee receives information about ACT from a neighbour who is also a health-care worker. The neighbour describes ACT as a drug combination that treats malaria efficiently and quickly without serious side effects. Also its relative high cost was explained. The TIOLI method was then used whereby the respondent was asked if he/she would buy ACT for TSh 500. If yes, the price was raised to TSh 1000 and then to TSh 2000 if accepting the previous level. Finally, after accepting to pay TSh 2000 or following a rejection of a bid, the respondent was asked to mention the maximum amount he/she was willing to pay for ACT. The levels of 500, 1000 and 2000 were selected to simplify the data collection and to clearly distinguish major differences in WTP. The follow-up question was included to provide an opportunity for the respondents to state their level of WTP more precisely.

Respondents willing to pay more than TSh 500 were assumed to afford ACT. In the multivariate analysis the respondents were classified into two groups to explore if associated characteristics influenced WTP: a) willing to pay less or equal to TSh 500 and b) willing to pay more than TSh 500 for ACT.

The economic level of the households was used as a local relative indicator of wealth among the included households. It was assessed by retrieving information from the heads using questionnaires on monthly expenditures on food, transport and half-yearly expenditures on health care, clothes and contributions. These areas of expenditure were selected based on information on the common local main expenditures items.

### Data analysis

The data was entered, prepared and analyzed by bivariate and multivariate approaches using the statistical computer programme SPSS. Logistic regression was used to analyze different variables' association with WTP. The dependent variable was WTP ≤ TSh 500 respective WTP > TSh 500. The independent variables were household status, occupation, education, household size and number of children. The results were expressed as proportions with 95% confidence intervals. Missing data and "no answer" and "do not know" were excluded.

In the analysis, each household was allocated to an economic quintile according to the average household expenditure per household member. To account for differences in household structure, the household expenditure was expressed as expenditure per adult equivalent (AE) [18].

AE = (A+αK)^θ^

A = Number of adults in the household.

K = Number of children in the household.

α = Cost of a child relative to that of an adult. According to Deaton and Zaidi (2002) this can be set to 0.3 for low-income countries.

θ = Controls the extent of economies of the scale. This can be set to 0.9 for low-income countries.

### Ethical clearance

Ethical clearance was obtained at the National Institute for Medical Research (NIMR), Dar es Salaam. Authorities at district and ward levels also provided approval for the study.

## Results

### Socio-demographic characteristics

A total of 269 adults, 135 mothers and 134 household heads, consented to the quantitative study whereas four were unwilling to be interviewed. Some 93 (69%) of the heads were male and 41 (31%) were female. Among the household heads, 90% were farmers and 77% had completed primary school and 19% secondary school. Among the mothers, 97% were farmers, 80% had completed primary and 18% secondary school. The median number of children in the interviewed households was 2, (range 1–6). A majority of the households included 5–8 household members.

### Willingness to pay for ACT

**Figure 1:**
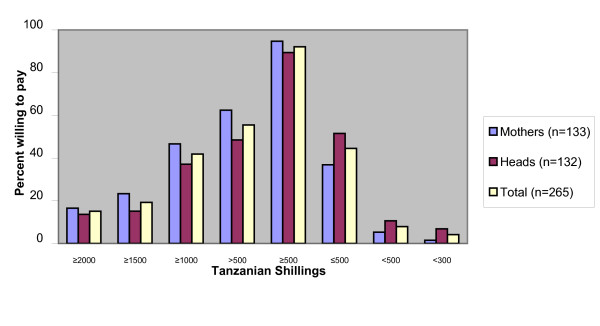
**Distribution of WTP for ACT among mothers and household heads, by price categories**. The statistical significant association with WTP was being a mother (unadjusted odds ratio = 2.0, CI = 2.1–3.4 and adjusted odds ratio = 2.1, CI = 1.2–3.6). The dependent variable in the calculation was WTP ≤ TSh 500 respective WTP > TSh 500. The independent variables were household status, occupation, education, household size and number of children.

Out of 265 respondents 244 (92%, 88%–95%) were willing to pay TSh 500 for ACT for a sick child in the household and 147 (55%, 49%–61%) were willing to pay more than TSh 500 (Figure 1). A total of 254 (96%, 93%–98%) were willing to pay TSh 300. The median amount that respondents were willing to pay was TSh 600.

WTP was not associated with education, occupation, household size or number of children under five. The only statistically significant association with WTP was being a mother (unadjusted odds ratio = 2.0, CI = 1.2–3.4 and adjusted odds ratio = 2.1, CI = 1.2–3.6). For the sake of methodological clarity mothers that were also household heads were excluded in this analysis. Little difference between the adjusted odds ratio (adjusted for the effect of the above mentioned variables) and the unadjusted odds ratio indicates that mothers' higher WTP is less likely to be due to confounding factors. Up to 72 (55%, 47%–64%) of the mothers are involved in the malaria treatment-decision for a sick child but only 22 (17%, 11%–24%) could also authorize the expenditure, either alone or together with the husband. Some 79% of the mothers reported that their husbands have to authorize the expenditure (Table 1).

**Table 1:** Decision-making on malaria treatment for a sick child under five as reported by mothers

	**Who decides to obtain an anti- malarial drug? **(n = 130)	**Who authorizes the expenditure? **(n = 130)
**Mother**	46 (35%, 28%–44%)	14 (11%, 7%–17%)
**Husband**	55 (42%, 34%–51%)	103 (79%, 71%–85%)
**Together**	26 (20%, 14%–28%)	8 (6%, 3%–12%)
**Relatives/others**	3 (2%, 1%–7%)	5 (4%, 2%–9%)
**Total**	130 (100%)	130 (100%)

The results are expressed with 95% confidence intervals.

Malaria was considered as the most serious disease in the community by 97 mothers (76%, 68%–82%). A total of 100 mothers stated that an anti-malarial drug is the best treatment for malaria, whereas 11 stated local herbs or antipyretics. Some 24 respondents did not provide an answer to this question or did not know. A total of 96 mothers (73%, 65%–80%) mentioned that mosquitoes are the cause of malaria. Almost half of the mothers (43%, 34%–51%) reported that someone in the household had suffered from malaria in the last two weeks. These measures were not, however, associated with WTP for ACT.

Household expenditures and assessment of previous failure to obtain malaria treatment were assessed from heads and compared with WTP. The household expenditure level, divided into quintiles, was not significantly related to the WTP for ACT (Table 2). However wide confidence intervals make the results uncertain. Up to 45 (34%, 27%–43%) of the respondents had failed access to malaria treatment due to lack of money and they were also less willing to pay for ACT (p = 0.033) (Table 2).

**Table 2:** WTP, expenditure and previous failure of accessing malaria treatment

**Expenditure quintiles (TSh)**	**0–2760**	**2761–4300**	**4301–5680**	**5681–7680**	**7681–18200**	**Access failure **	**No access failure**
**WTP ≤ TSh 500**	11 (52%, 32%–72%)	11 (52%, 32%–72%)	11 (50%, 31%–69%)	10 (45%, 27%–65%)	10 (48%, 28%–68%)	29 (64%, 50%–77%)*	39 (45%, 35%–55%)

**WTP > TSh 500**	10 (48%, 28%–68%)	10 (48%, 28%–68%)	11 (50%, 31%–69%)	12 (55%, 35%–73%)	11 (52%, 32%–72%)	16 (36%, 23%–50%)	48 (55%, 45%–65%)

**Total**	21 (100%)	21 (100%)	22 (100%)	22 (100%)	21 (100%)	45 (100%)	87 (100%)

WTP in relation to levels of expenditure (n = 107, p = 0.98) and previous failure of accessing malaria treatment (n = 132). Data was obtained from household heads. Results are expressed with 95% confidence intervals. (*) p = 0.033

### Ability to pay for ACT

A child in the rural area of the study was assumed to need an ACT four times yearly and an adult once yearly [19,20]. The average price of an ACT was assumed to be US$ 1.0 per adult treatment dose and US$ 0.5 per child treatment dose, based on information from Novartis and Sanofi-Aventis [10,11]. Hence, the annual treatment cost would average US$ 2.0 for a child and US$ 1.0 for an adult. These costs were then multiplied by the number of adults and children respectively in each study household. Accordingly, a non-subsidized median household expenditure for ACT was calculated at US$ 6.0. In the study area, the cost of the introduced ACT in 2008 (Coartem^®^) was provided free of charge for children and at a cost of US$ 0.25 for an adult dose. This would imply an annual median household expenditure of US$ 0.75.

**Figure 2:**
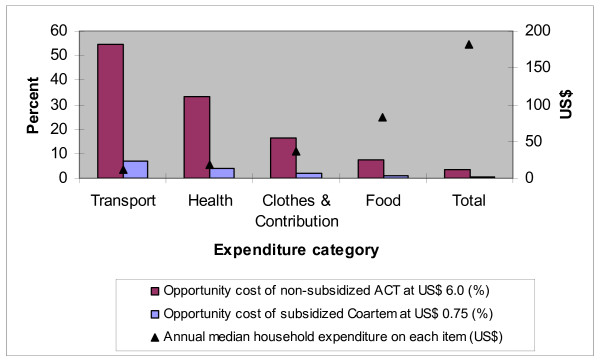
**Comparison of the opportunity costs of ACT**. The calculated annual median household expenditure for non-subsidized ACT at US$ 6.0 is compared with the current (2008) local annual median household expenditure for subsidized Coartem^® ^at US$ 0.75. The opportunity cost is expressed as percentage of five expenditure categories (n = 115).

Comparisons were made of the opportunity costs of a) unspecified non-subsidized ACT and b) subsidized Coartem^®^. The annual median household expenditures for health care, food, clothes & contributions, local transport and all items taken together (US$ 182, range 28–1010) were compared to these numbers. The opportunity cost was expressed as percentage of the five expenditure categories (Figure 2). The opportunity costs of non-subsidized ACT and subsidized Coartem^® ^on the reported total annual expenditure on food were 7.0% and 0.9% respectively. The opportunity costs on the reported total annual expenditure on health care were 33.0% and 4.2% respectively.

According to official data [21], the average annual rural household consumption expenditure in Tanzania was US$ 636 [US$ 1 = TSh 876 [22]) in 2001. The median annual household cost for ACT (US$ 6.0) represents 0.9% of this. The median annual household cost of subsidized Coartem^® ^(US$ 0.75) represents 0.1% of this.

### Perceptions and desired features of anti-malarial drugs

The general opinion about Fansidar, assessed through focus group discussions and in-depth interviews with 24 respondents, was that the disadvantages outweigh the advantages. The main objections to Fansidar were poor curative effect and an adverse effect, described as "hangover". This implies extra expenses such as purchase of juice, which can relieve this adverse effect. A "short regimen", "few tablets" and "inexpensive" were positive comments about Fansidar. A vast majority of the respondents do think there is a need for a better anti-malarial drug than Fansidar. The same respondents were then asked if they had heard about combination therapy for malaria (dawa ya mseto) or anti-malarial drugs containing artemisinin (Artemedine^®^, Coartem^®^). Two of the 24 respondents had heard about ACT/artemisinin drugs.

**Figure 3:**
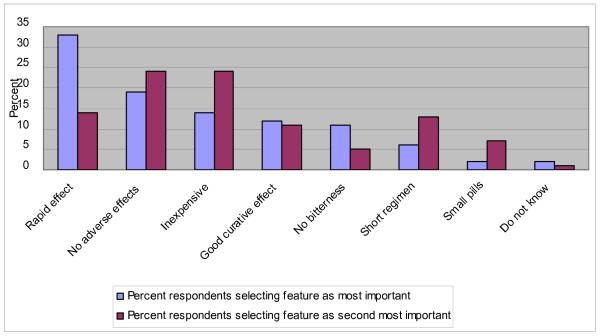
**Most desired features of an anti-malarial drug according to mothers and heads**. The respondents were presented with a number of features of a hypothetical drug and were asked to quote features in orders of importance for frequent use in the household (n = 245).

Mothers and heads (n = 245) were presented with a number of features of a hypothetical anti-malarial drug and were asked to quote features in order of importance for frequent use (Figure 3). "Rapid effect" was quoted as most important (33%, 28%–40%) followed by "no adverse effects" (19%, 15%–25%) and "inexpensive" (14%, 10%–19%). "Inexpensive" was quoted by 62% (56%–68%) as at least one of three important features.

There was no statistically significant correlation between the "most important" feature selected by the respondent and the household status, occupation or education. Heads of households with an average or below average expenditure level, however, mentioned "inexpensive" as an important feature more frequently than those with above average expenditure level (68% compared to 44%, p = 0.021).

## Discussion

Only half of the mothers and household heads were willing to pay more than TSh 500 for a child's dose of ACT and likely to be willing to pay US$ 6.0 per year, the median estimated household cost for non-subsidized ACT. However, 96% were willing to pay TSh 300.

In 2007 Tanzania changed its malaria treatment policy to make Coartem^® ^the first-line treatment for uncomplicated malaria. For a three-year period it will be provided free to all at governmental health care facilities through the Global Fund support and be available for a cost of TSh 300–500 (US$ 0.28–0.46) at specially designated shops through the programme of Accredited Drug Dispensing Outlets (ADDOs). In the study area Coartem^® ^was thus introduced in 2007, free for children below five years of age but at a subsidized consumer cost of US$ 0.25 for an adult dose.

The decisions to subsidize to the above levels in the public health facilities and ADDOS appear to be well aimed, since they cover almost all households' WTP for ACT. However, the present policy may not continue after the introductory phase. External funding is likely to be needed in order for malaria interventions to be effectively implemented and sustained. This finding supports earlier studies including other malaria control measures [12,13,23].

Mothers showed a higher WTP for ACT than male household heads. To what extent the mothers' higher WTP will influence the decision behaviour is unclear. A vast majority of authorizations of treatment-decisions for the children are made by their husbands. This may however conceal a complex process of decision-making within the family before the household head's ultimate decision.

The median level of WTP of TSh 600 in the present study without prior knowledge of ACT is in line with an earlier study in Tanzania where mothers' WTP for ACT was TSh 650 to 750 after experiencing treatment of ACT to their children [13].

Contingent valuation studies are criticized for being too hypothetical and generating results with low validity, since results depend on the choice of eliciting method. The hypothetical scenario in this study was aimed at being easily understood by the respondent and as realistic as possible, although ACT was unknown to people prior to the study. TIOLI was considered the most appropriate eliciting method since the malaria treatment usually is not subject to bargaining but has the character of "buy it or leave it". A main concern attributed to the CV method is that the elicited monetary value correlates poorly with reality, since respondents are subjected to a hypothetical situation wherein their WTP is assessed. When hypothetical WTP has been compared to actual WTP, a divergence between the hypothetical WTP and purchase behaviors has been demonstrated [24-27]. Suggested factors behind this divergence have been measurement biases overestimating WTP, and legitimate changes in valuation with time [16,28]. When the commodity is important to the respondents, the discrepancy between hypothetical and actual WTP may be smaller [29].

Measurement bias may have resulted from weak understanding of the questions. To improve the understanding, the interviewers prepared explanations of questions in Kihaya, although people in the area are often quite familiar with both Kiswahili and Kihaya. This may have influenced the wording of the questions but with a purpose of increasing understanding for some respondents.

WTP is normally related to ATP [30]. However, WTP may not directly reflect ATP in developing countries because mobilization of resources in order to pay for health care may require that basic needs such as food and education are sacrificed with serious consequences for the households [31]. The expenditure level of households did not correlate with WTP in this study. That could be due to possible measurement problems of this variable caused by poor recall or that the economic differences between the respondents were not prominent when using the included expenditure categories. Including total food expenditure, school-fees, household equipment and housing may have been better for describing the economic differences between the households. However, there was similarly no correlation between WTP and the socio-economic status in the previous study from Tanzania [13].

There is a large discrepancy between the total annual median expenditure estimated in the present study (US$ 182) and the official data (US$ 636). Excluded expenditure items in this study like school-fees, housing and household equipment as well as the benefit of food produced by the household in this fertile area, may explain some of the discrepancy.

WTP was significantly correlated with previous failure of accessing malaria treatment. "Previous failure of accessing malaria treatment" may represent a better variable than "expenditures" for estimating the ATP. It might be easier to remember and report than expenditures. It may also be more socially desirable to describe oneself as having poor financial access to drugs than having low economic status.

It has been pointed out that the tendency to provide answers perceived to be the desired response, social desirability bias, should be considered in WTP studies [32]. Other studies have found that the contingent valuation method may be influenced by the fact that respondents deliberately understate their WTP to influence the price of a new product, so called strategic bias [33]. However, in the present study, respondents had little prior knowledge of ACT and probably did not see any gain in projecting low WTP for it.

Among desired features of an anti-malarial drug, the respondents considered "rapid effect" and "no adverse effects" more important than "inexpensive" or "good curative effect". This suggests that recurrence of malaria may not be perceived as an indicator of ineffective treatment and that the immediate relief of symptoms is most important.

The median household cost of an ACT as 0.9% of the total annual average household expenditures in Tanzania would in a rich country like Sweden represent US$ 332, as estimated from official data from 2001 [21,22,34]. Subsidized Coartem's^® ^annual median household cost as 0.12% of the total annual average household expenditures would represent US$ 42.

## Conclusion

Small fluctuations in price may have substantial impact on WTP and treatment seeking behaviour. Male heads of households are less willing to pay than the caregivers of children. This is to be considered when designing suitable financial strategies for making first-line anti-malarial drugs available to families and when sensitising people with information on recommended malaria treatment.

The upper limit of the price for a child's dose of ACT should not exceed Tsh 500 (US$ 0.46), so as to be financially accessible and used in this community. Subsidies are, therefore, needed to make ACT affordable to the average households in this and other low-income settings. This study also shows that before the introduction of ACT as first-line treatment, there was a wish and need for a new, better, affordable and reliable anti-malarial drug.

The present study, however, generally supports the decision to subsidize Coartem^® ^to a consumer price of TSh 0–500 as it appears to cover almost all households' WTP for ACT. Since malaria control has been proven to have high cost-effectiveness [35], both national and external funding should, therefore, ensure that the present policy of ACT subsidy is sustained.

## Competing interests

The authors declare that they have no competing interests.

## Authors' contributions

ECS initiated the study and was actively engaged in all stages of the work from study design to completion of the manuscript. BCF made considerable contribution to interpretation of study findings and work on the manuscript and revised it critically for substantial intellectual content. ZP made substantial contributions to conception and design of the study. SMM critically edited the manuscript and provided advice on the method and interpretation of results. AB supervised the work from its onset and contributed with continuous input in work from study design to finalization of the manuscript.
